# Differences in susceptibility and immune responses of three-spined sticklebacks (*Gasterosteus aculeatus*) from lake and river ecotypes to sequential infections with the eye fluke *Diplostomum pseudospathaceum*

**DOI:** 10.1186/1756-3305-7-109

**Published:** 2014-03-21

**Authors:** Jörn Peter Scharsack, Martin Kalbe

**Affiliations:** 1 Department of Evolutionary Ecology, Max Planck Institute of Evolutionary Biology, August-Thienemann Str 2, Plön 24306, Germany; 2 Department of Animal Evolutionary Ecology, Institute for Evolution and Biodiversity, University of Münster, Hüfferstr 1, Münster 48149, Germany

## Abstract

**Background:**

The eye fluke *Diplostomum pseudospathaceum* is a frequent parasite of many fresh-water fish species, among those three-spined sticklebacks, particularly in lakes with lymnaeid snails, its first intermediate hosts. Cercariae released from host-snails, penetrate the skin of their fish hosts and within 24 h migrate to the immunologically inert eye lenses. Thus, individual *D. pseudospathaceum* are exposed to the fish immune system only for a short time, suggesting that only innate immunity can be active against the parasite. However, in nature sticklebacks are exposed to *D. pseudospathaceum* repeatedly since snails are shedding cercariae from late spring to autumn. Therefore, acquired immunity after initial infection would be advantageous against subsequent parasite encounters.

**Methods:**

We investigated if sticklebacks originating from a lake with high and from a river with low prevalence of *D. pseudospathaceum* differ in susceptibility to repeated exposure to the parasite. We compared infection success and immune functions in laboratory-bred sticklebacks from both habitats in naïve fish with fish that had been pre-exposed to eye flukes. Head kidney leukocytes (HKL) from experimental sticklebacks were investigated for respiratory burst activity and the proliferation of lymphocytes and monocytes 1.5, 5 and 15 days after infection.

**Results:**

Lake sticklebacks were less susceptible than river sticklebacks, however, in both populations pre-exposure led to a similar relative reduction in infection success. The respiratory burst activity was higher with HKL from lake sticklebacks and was up-regulated in pre-exposed fish but dropped 1.5d after an additional exposure, suggesting that activation of phagocytic cells is crucial for the defense against *D. pseudospathaceum*. Changes in lymphocyte proliferation were only detectable 1.5d after the last exposure in lake sticklebacks, but not 5 and 15d post exposure, indicating that a lymphocyte mediated acquired immune response was not induced. Proliferation of monocytes was significantly increased 1.5d after the last exposure with HKL from both stickleback populations.

**Conclusions:**

Increased resistance to *D. pseudospathaceum* in sticklebacks from both populations upon pre-exposure cannot be explained by a prominent adaptive immune response. Monocytic leukocytes were more responsive, suggesting that rather cells of the innate than the adaptive immune system are active in the defense of *D. pseudospathaceum*.

## Background

The eye fluke *Diplostomum pseudospathaceum* is a frequent parasite of many fish species. Cercariae of *D. pseudospathaceum*, hatched from lymnaeid snails, penetrate through the skin of their fish hosts and within 24 h migrate to the immunologically inert eye lenses [1]. Here the parasite grows until the fish is preyed upon by its final bird host [2,3]. In the fish eye lenses, the parasites cause cataracts, which reduce the hosts visual abilities [4,5] and are likely predisposing fish to predation by the final host [6]. Therefore, selection pressure on host immunity, to defend the infection is high.

Eye fluke parasites such as *D. pseudospathaceum*, are exposed to the immune system of their fish hosts only for a relatively short time, before they hide in the immunologically privileged eye lens [7]. In the wild, repeated exposure of individual hosts to infectious stages (cercariae) of eye flukes is likely and hosts would benefit from acquired immunity. Here we test if three-spined sticklebacks (*Gasterosteus aculeatus*), a typical host of *D. pseudospathaceum*, are capable to acquire immunity against the eye fluke after repeated exposure.

In three-spined stickleback naturally infected with *D. pseudospathaceum* considerable differences of the parasites prevalence across stickleback populations was observed [8]. This depended on the habitat type and in river populations the prevalence of *D. pseudospathaceum* was low, due to lower abundance of the first intermediate snail hosts, while in lakes prevalence of the parasite was high [8,9].

In the investigated area (Northern Germany), sticklebacks from lake and river populations have been shown to be genetically distinct ecotypes [10]. In the lakes, sticklebacks are adapted to higher frequency and diversity of parasites, which resulted in higher resistance to parasite infections compared to river sticklebacks [11-13]. In a field enclosure in a lake, with naturally occurring parasites, lake sticklebacks accumulated less parasites compared to river sticklebacks [13]. After experimental exposure to the typical lake parasite *D. pseudospathaceum*, lake sticklebacks showed lower infection rates compared to river sticklebacks [11,12]. This suggests that stickleback ecotypes adapt their immunocompetence to the parasites present in their habitat type of origin.

The underlying immunological prerequisites are only partly investigated yet. Leukocytes isolated from naïve and *D. pseudospathaceum* exposed lake sticklebacks exhibited higher respiratory burst activity than leukocytes from river sticklebacks [11]. The expression of immune genes, predominantly genes of innate immunity, was higher in lake compared to river sticklebacks after exposure to *D. pseudospathaceum* (in combination with two other sympatric parasites) [12].

In the study by Kalbe and Kurtz [11] pre-exposure of both stickleback ecotypes to *D. pseudospathaceum* reduced the infection success of a later exposure only marginally, suggesting that acquired immunity is activated by the infection to a minor extent only. However, in this experiment sticklebacks were pre-exposed only two times with 20 cercariae each, which might not have been sufficient to induce substantial acquired immunity.

Rainbow trout (*Oncorhynchus mykiss*) are capable of behavioral avoidance of *D. spathaceum* infections [14] and are acquiring significant but not complete resistance to the parasite after being repeatedly pre–exposed [15-17]. Rainbow trout react with a specific antibody response against diplostomulae, the tissue-migrating stage of eye flukes [18,19] and are able to mount a cellular reaction [20,21]. The immune protection mechanism in rainbow trout against *D. spathaceum* involves both the alternative and the classical pathways of complement activation [20,22]. However, it seems that primarily non-specific responses protect against challenge infections with *D. spathaceum* cercariae [17]. It was observed that *O. mykiss* infected with live cercariae of *D. spathaceum* did not produce an antibody response, but fish injected with frozen cercariae, diplostomulae or metacercariae, displayed significantly higher levels of specific antibody than control fish [17]. This suggests that *Diplostomum* spp. are able to evade the specific antibody response, when they use their natural infection route, through the skin and via the blood to the eye lens. This might be facilitated by the relatively short time (approx. 24 h) during which the diplostomulae are actually exposed to the immune system, before they reach the lenses.

**Figure 1:**
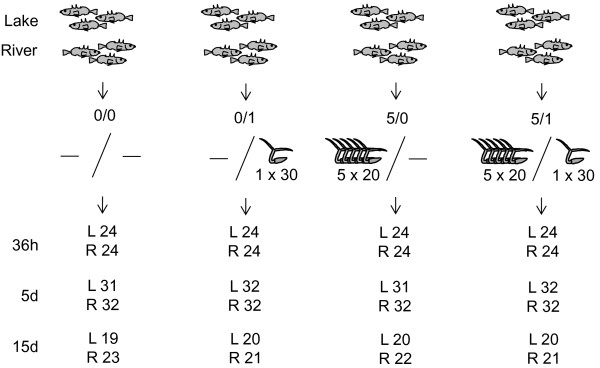
**Experimental set up.** Sticklebacks were laboratory offspring of a lake and a river population (4 families each, 70–80 individuals per exposure setup). Sticklebacks were sham exposed (0/0), exposed 1 × with 30 (0/1), 5 × pre-exposed with each 20 (5/0) and 5 × pre-exposed with each 20 and 1 × exposed with 30 (5/1) *D. pseudospathaceum* cercariae. Sticklebacks were investigated for small metacercariae from the last exposure, large metacercariae from the pre-exposures and immune parameters 36 h, 5 days and 15 days after the last exposure with n = 19–32 individuals per treatment group (L = lake, R = river).

However, acquired immunity of fish to *Diplostomum* spp. infections was observed, which might be based mainly on mechanisms of innate immunity. We hypothesized that sticklebacks originating from a lake, which are more resistant to *D. pseudospathaceum*, might also be capable to acquire higher resistance to the parasite upon pre-exposure, compared to river sticklebacks, which are more susceptible to *D. pseudospathaceum*. To test this hypothesis laboratory offspring of a lake and a river stickleback ecotype were five times pre-exposed to *D. pseudospathaceum* and singly exposed thereafter with the respective controls (sham exposed, pre-exposed only, and singly exposed only, Figure 1). In addition to the infection success of *D. pseudospathaceum* we analyzed the respiratory burst activity of stickleback head kidney leukocytes as a parameter of innate immunity and lymphocyte proliferation to detect a potential acquired immune response.

## Methods

### Experimental sticklebacks and parasites

Three-spined sticklebacks (*Gasterosteus aculeatus*) were caught from a river (Malenter Au, 54°12′16.19″N, 10°33′32.93″E) and a lake (Großer Plöner See, 54°9′21.16″N, 10°25′50.14″E), belonging to the same drainage systems in northern Germany in autumn 2006. Four crosses between wild caught fish from each of the two populations were produced, as described elsewhere [11]. Hatched offspring of each family were kept under summer conditions (18°C and a 16 h/8 h light/dark rhythm) in 16 L tanks with a continuous flow through of 2 L/h for the first two months. Here they were fed with live *Artemia salina*. Later, fish were kept at a density of 20 fish per tank and fed with frozen chironomid larvae. After seven months of growth in summer conditions at a density of 20 fish per tank, fish were used for the experiment.

*Lymnaea stagnalis* snails were collected in another lake (Kleiner Plöner See, 54°9′42.15″N 10°22′43.31″E) directly connected to and in a distance of 3.3 km from the origin of the lake stickleback population used for the experiment. Snails were checked for patent infections with *Diplostomum pseudospathaceum* and kept in tanks under the same conditions as the sticklebacks and were fed with washed lettuce *ad libitum*.

### Parasite exposure and dissection protocol

An overview of the parasite exposure set-up is given in Figure 1. Experimental fish from four lab-bred stickleback families of each of the two habitat types were divided into four groups and kept in 16 L tanks. Where possible these four groups consisted of 20 fish. However, in two of the lake and one river families less than 80 fish were available (instead only 66, 76 and 77, respectively) and the group sizes had to be reduced accordingly. Two of the four tanks from each family where then assigned as the pre-exposed group. Prior to each round of pre-exposure the water flow was switched of and the volume in the tanks was decreased to 10 L. The infective cercariae were obtained by placing *L. stagnalis* snails naturally infected with *D. pseudospathaceum* individually in glass beakers with 100 ml of water under bright illumination for 2 h. Thereafter, equal amounts of cercariae released from 10 snails where pooled, as described in Kalbe & Kurtz [11] and the density of parasite larvae was determined. From this pool the volume corresponding to 20 cercariae per fish were added to the tanks with the pre-exposure groups. This kind of semi-controlled mass infection was repeated every two weeks, five times in total, whereas the tanks with the fish serving as controls and for the single exposed groups where equally treated, but just received the equivalent volume of pure water instead of the cercarial suspension. The water flow in the tanks was switched on again only after 24 h to ensure that cercariae were not washed out the tank within their short lifespan.

For the final experimental infection, two weeks after the fifth pre-exposure, fish from all treatment groups were placed individually in small tanks containing 1 L of water. This time, however, cercariae were individually counted and each fish of the ‘singly exposed’ (0/1) and the ‘pre-exposed + singly exposed’ (5/1) group received 30 cercariae, which were added to the tanks in Petri dishes 2 h after they had been shed from the snails and pooled accordingly. Sham exposed controls (0/0) and ‘pre-exposed only’ (5/0) fish again received pure water instead of cercarial suspension.

The fish were left in the infection tanks for 36 h before the first cohort of fish were dissected, whereas the groups to be dissected 5 days and 15 days post exposure (plus the respective control fish) were transferred back into the common 16 L aquaria.

For dissection, fish were stunned by a blow on the head, weighed (to the nearest 0.1 mg) and the total length measured (to the nearest mm), before they were killed by decapitation. Then the body cavity was opened and the head kidneys were removed completely and immediately placed on ice before processed further for immunological assays. Infection rates of *D. pseudospathaceum* were determined by carefully dissecting the eye lenses, in which all large (from the pre-exposed treatment) and small metacercariae (final experimental infection) were counted. The discrimination of parasites from the different treatment by size was possible only in the cohorts dissected after 36 h and 5 days, but not in the group processed after 15 days. The whole experiment was performed in two independent rounds with a time lag of one week, in order to be able to follow the strict time schedule.

### Isolation of head kidney leukocytes

For immunological assays, leukocytes were isolated from the head kidney of sticklebacks. All steps for leukocyte preparation were performed on ice and only refrigerated media and cooled centrifuges were used. Cell suspensions from head kidneys were prepared by forcing the tissues through a 40 μm nylon screen (BD-Falcon, USA). Isolated head kidney leukocytes (HKL) were washed twice (4°C, 10 min 550 × g) with RPMI 1640 diluted with 10% (v/v) distilled water (R-90). Numbers of viable cells (exclusion of propidium iodide positive cells) were enumerated by means of flow cytometry.

### Flow cytometric analysis of freshly isolated head kidney leukocytes

Total cell numbers were determined with the standard cell dilution assay (SCDA) [23] in a modified form [24]: washed cells were transferred to individual wells of 96 well round bottom plates; 2 × 10^4^ green fluorescent standard particles (4 μm, Polyscience, USA) and propidium iodide (2 mg L^−1^, Sigma Aldrich) were added to each well. FSC/SSC characteristics of at least 10,000 events were acquired in linear mode; fluorescence intensities at wavelengths of 530 nm and 585 nm were acquired at log scale with a flow cytometer (FACSCalibur, Becton and Dickinson, USA) with an automated sampling unit for 96 well plates. Flow cytometric data were analysed with the CellQuest Pro 4.02 software for acquisition and analysis. Cellular debris with low FSC characteristics was excluded from further evaluation. Standard particles (green fluorescence positive) were discriminated from viable HKL (propidium iodide-negative, green fluorescence negative). Absolute numbers of cultivated cells in individual wells were calculated according to: N [vital cells] = Events [vital cells] × Number [standard beads] / events [standard beads].

### Respiratory burst activity of head kidney leukocytes

As one of the most important effector mechanisms of the innate immune system, the respiratory burst activity of head kidney leukocytes (HKL) was quantified in a lucigenin-enhanced chemiluminescence assay modified after Scott and Klesius [25], as described by Kurtz *et al.*[26]. In 96 well flat bottom micro titre plates, 160 μl of cell suspension (1 × 10^5^ HKL/well) were added to 20 μl lucigenin solution (2.5 g L^−1^ PBS) in two replicates per stickleback. Plates were incubated for 30 min at 18°C to allow uptake of lucigenin by the cells. One of each HKL replicate received 20 μl zymosan suspension (7.5 g L^−1^ PBS) and the other 20 μl *D. pseudospathaceum* antigens (40 mg L^−1^ PBS, prepared as described by Hibbeler *et al.*[27]) to induce production of reactive oxygen species (ROS). The chemiluminescence was measured with a micro titre plate luminometer (Berthold, Germany) for 3 h at 18°C. Relative luminescence (RLU) was calculated for each sample using the WinGlow software.

### Cell cycle analysis

As a parameter for activation of the adaptive immune system, we determined the relative number of lymphocytes in the S and G2M phase of the cell cycle after DNA labelling with propidium iodide by means of flow cytometry.

Head kidney leukocytes were fixed with ethanol (100 μl cell suspension as described above in 900 μl ice cold Ethanol 98%) and stored at 4°C. For cell cycle analysis, cells were centrifuged (550 × g, 10 min, 4°C) and supernatant ethanol was removed. Cells were resuspended with RNAse (500 mg L^−1^ PBS) and incubated for 10 min at room temperature to remove background labelling of RNA. Propidium iodide (Sigma Aldrich) was added to a final concentration of 7.5 mg L^−1^ and cells were incubated again for 10 min at room temperature. For individual samples, events were measured for three minutes or up to 30000 events with a Becton Dickinson FACSCalibur flow cytometer. Red fluorescence (propidium iodide) was measured in linear mode. Data were evaluated with the Cellquest Pro 4.02 software. Cellular debris (low scatter characteristics) and aggregated cells (high scatter characteristics) were subtracted from further evaluation. Doublet cells were subtracted from single cells as described by [28]. Lymphocytes and monocytes were identified according to their characteristic FSC/SSC profile. Frequencies of lymphocytes and monocytes in G_0–1_, S and G_2-M_ phase were acquired by DNA content analysis of red fluorescence intensity (propidium iodide labeling) of single cells from the respective gate.

### Statistical analysis

Statistical analyses were performed with the SPSS v 20 software (IBM, USA). Data were tested for normality with the Kolmogorov-Smirnov Test and by visual examination of histograms. Data were box-cox transformed if normality was not achieved. Homogeneity of variance was tested with the Levene’s Test. Effects of main factors (stickleback origin, parasite exposure treatment, sampling time point) and their interactions on response variables were analysed with Generalized Linear Models (GzLM) with stickleback family nested in stickleback origin (lake/river). Multiple pairwise comparisons were analysed with post hoc tests and sequential Bonferroni correction for multiple testing.

### Ethical note

Sticklebacks were maintained and treated in accordance with the local animal welfare authorities and the EU Directive 2010/63/EU for animal experiments. All animal experiments described were approved by the ‘Ministry of Energy, Agriculture, the Environment and Rural Areas’ of the state of Schleswig-Holstein, Germany (reference number: V 313–72241.123-34).

## Results

### Infection success

**Figure 2:**
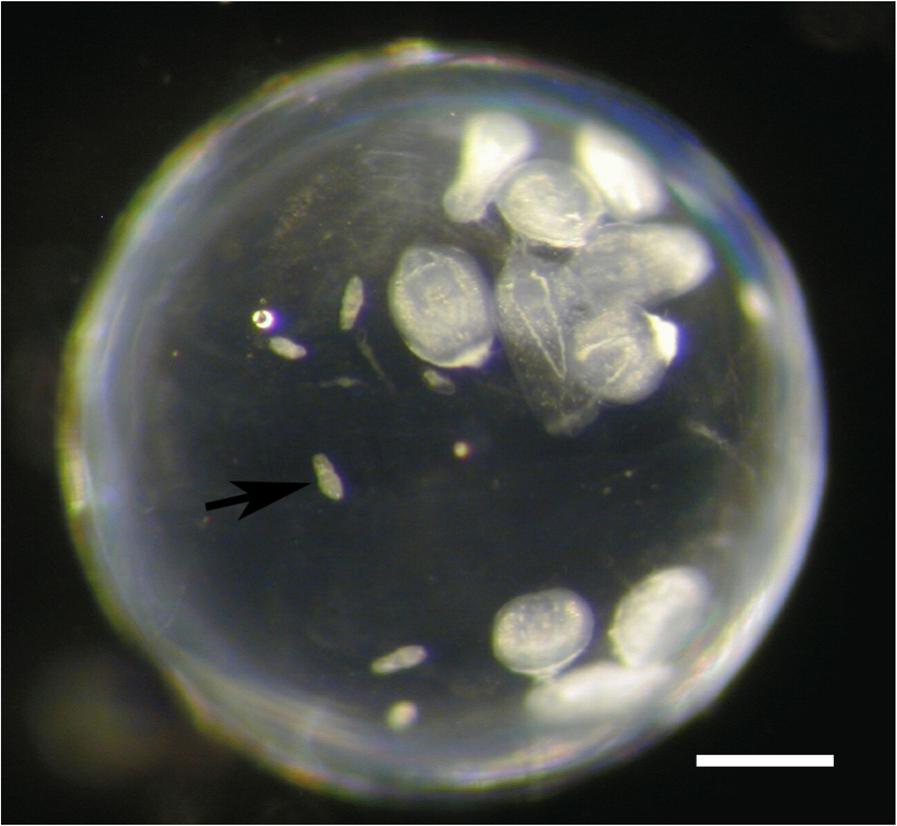
**Eye lens dissected from a lab bred three-spined stickleback after repeated experimental infection with *Diplostomum pseudospathaceum*.** Larger metacercariae originate from an exposure several weeks before, whereas the small larvae (arrow) are less than 40 h old (bar 200 μm).

**Figure 3:**
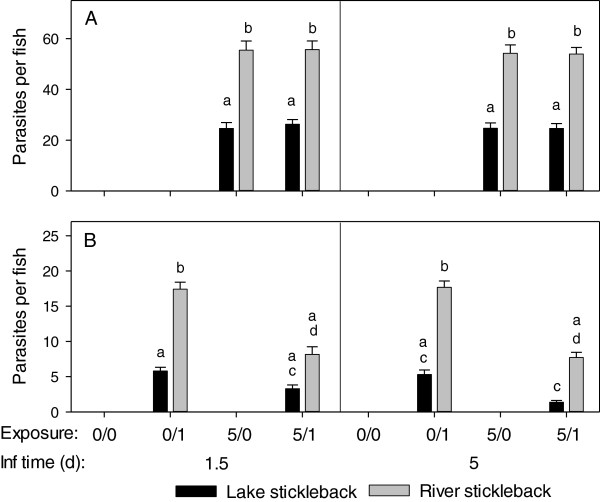
**Infection success of *D. pseudospathaceum*.** Eye flukes from pre-exposures **(A)** were larger and thereby discriminated from those of the single exposures **(B)** 1.5 and 5 days after the last exposure (mean and SE, 0/0 = sham exposed controls, 0/1 = single exposure only, 5/0 = pre-exposures only, 5/1 = pre- and single exposures, different letters above bars indicate significant differences p < 0.05 with sequential Bonferroni correction for multiple testing). Note: In all exposure setups, lake sticklebacks (black) had significantly less parasites than river sticklebacks (grey) at both time points. Single exposure of naïve sticklebacks (0/1) resulted in significantly higher numbers of parasites per fish compared to pre-exposed sticklebacks (5/1).

**Figure 4:**
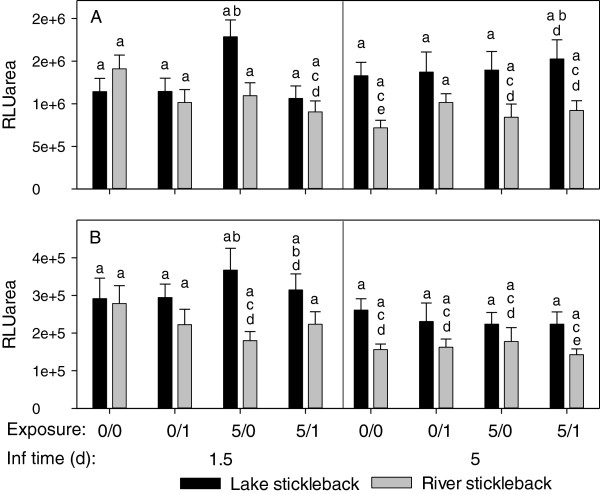
**Respiratory burst activity in *D. pseudospathaceum *exposed sticklebacks.** Head kidney leukocytes were stimulated with zymosan **(A)** and *D. pseudospathaceum* antigens **(B)**. Respiratory burst activity was recorded with a chemiluminescence assay (RLUarea) 1.5 and 5 days after the last exposure (mean and SE, 0/0 = sham exposed controls, 0/1 = single exposure only, 5/0 = pre-exposures only, 5/1 = pre- and single exposures, different letters above bars indicate significant differences p < 0.05 with sequential Bonferroni correction for multiple testing). Note: different y-axis scaling in **A** and **B**.

**Figure 5:**
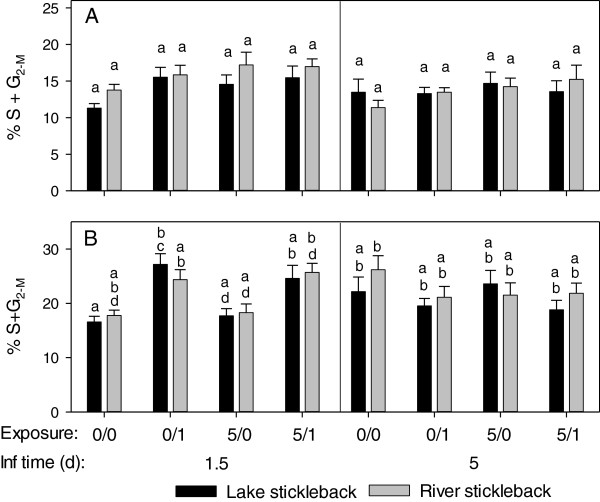
**Proliferation of lymphocytes (A) and monocytes (B) in *D. pseudospathaceum *exposed sticklebacks.** Head kidney leukocytes were subjected to cell cycle analysis by means of flow cytometry 1.5 and 5 days after the last exposure. Proportions of cells in the S and G_2-m_ phase of the cells cycle were determined in the lymphocyte and the monocyte gate (mean and SE, 0/0 = sham exposed controls, 0/1 = single exposure only, 5/0 = pre-exposures only, 5/1 = pre- and single exposures, different letters above bars indicate significant differences p < 0.05 with sequential Bonferroni correction for multiple testing). Note: different y-axis scaling in **A** and **B**.

In the eye lenses of sticklebacks exposed to *D. pseudospathaceum*, metacercariae from pre-exposures had larger body size (Figure 2) and could be discriminated from metacercariae from the single exposure 1.5 and 5 days after the last exposure (Figure 3), but not 15 days post last exposures. Therefore data from the 15 days sampling time point were excluded from the statistical analysis. In the generalized linear model (GzLM) analysis of the infection data, origin of the stickleback hosts (lake/river) and stickleback family nested in origin had strong effects on the infection success of *D. pseudospathaceum* (Table 1). The numbers of metacercariae per eye lens did not change significantly between sampling time points (Table 1), as we expected. Nevertheless we blotted the infection data for both time points (Figure 3) for comparison with the immune data (Figures 4 and 5). Overall, river sticklebacks had higher numbers of parasites compared to lake sticklebacks (Figure 3). This was significant for metacercariae from pre-exposures (Figure 3A) as well as metacercariae from single exposures (Figure 3B) (Table 1) at both sampling time points. The GzLM revealed that the exposure treatment influenced the infection success significantly only of young diplostomules. Numbers of these metacercariae per fish, from the recent single exposure of naïve sticklebacks (0/1, Figure 3B) were higher compared to pre-exposed sticklebacks (5/1, Figure 3B), significantly only for river sticklebacks. This indicates that sticklebacks upon (previous) exposure to the parasite become more resistant to subsequent infection. Total counts of parasites from single exposures were again significantly lower in lake sticklebacks, but the relative decrease in the number of successful parasites upon pre-exposure was similar in lake (60.2%) and river (55.0%) sticklebacks.

**Table 1:** **Main effects of the statistical models of the infection success of *D. pseudospathaceum***

		**Old diplostomules**		**Young diplostomules**	
	**df**	**χ**^**2**^	**P value**	**χ**^**2**^	**P value**
Intercept	1	2144.438	< 0.001	1225.4	< 0.001
Family(origin)	6	46.261	< 0.001	43.8	< 0.001
Sampling time point	1	0.404	0.525	2.2	0.137
Origin	1	297.3	< 0.001	348.8	< 0.001
Exposure treatment	1	0.038	0.846	178.8	< 0.001
Samp. t. x orig. x exp. treat.	4	0.234	0.994	50.3	< 0.001

Older diplostomules from pre-exposures (in n = 223 sticklebacks) were larger and thereby discriminated from young diplostomules (in n = 224 sticklebacks, compare Figure 1) from the single exposures 1.5 and 5 days after the last exposure (compare Figure 3). Data were analysed with Generalized Linear Models (GzLM) with stickleback family nested in stickleback origin (lake/river).

### Respiratory burst activity of head kidney leukocytes

The respiratory burst activity of head kidney leukocytes (HKL) across treatments and stickleback origins was higher in zymosan stimulated cultures, compared to stimulation with *D. pseudospathaceum* antigens (Figure 4A,B). Stickleback origin (lake/river) and family nested in origin significantly affected the respiratory burst response to both, zymosan and *D. pseudospathaceum* antigens (GzLM, Table 2). Head kidney leukocytes (HKL) from river sticklebacks showed generally lower respiratory burst activity than HKL from lake sticklebacks, most evidently 5 days after the last exposure (Figure 4A,B).

**Table 2:** **Main effects of statistical models of the immune parameters of *D. pseudospathaceum *** **exposed sticklebacks**

		**RLUarea zymosan**		**RLUarea *D. p. *** **antigens**		**% S + G**_**2-m **_**lymphocytes**		**% S + G**_**2-m **_**monocytes**	
	**df**	**χ**^**2**^	**P value**	**χ**^**2**^	**P value**	**χ**^**2**^	**P value**	**χ**^**2**^	**P value**
Intercept	1	882.0	< 0.001	750.1	< 0.001	1880.4	< 0.001	2249.9	< 0.001
Family(origin)	6	49.8	< 0.001	45.4	< 0.001	8.1	0.232	9.6	0.145
Sampling time point	1	0.5	0.501	18.6	< 0.001	4.9	0.028	0.2	0.632
Origin	1	20.6	< 0.001	23.7	< 0.001	1.5	0.220	1.1	0.291
Exposure treatment	3	2.9	0.401	0.98	0.804	10.7	0.013	7.2	0.065
Samp. t. x orig. x exp. treat.	10	23.4	0.009	7.2	0.705	7.1	0.716	41.8	< 0.001

Respiratory burst activity (RLUarea) was recorded with zymosan and stimulation with *D. pseudospathaceum* antigens (Figure 4), and proliferation (% S + G_2-m_) of lymphocytes and monocytes (Figure 5) was recorded from n = 446 sticklebacks (Figure 1). Data were analysed with Generalized Linear Models (GzLM) with stickleback family nested in stickleback origin (lake/river).

Differences in the respiratory burst between exposure groups within stickleback origin lake/river and sampling time point were not significant with Bonferroni correction, however, highest respiratory burst was observed with HKL from lake sticklebacks with pre-exposures only (5/0), but this was only significant compared to river sticklebacks with pre- and recent exposure (5/1) in zymosan stimulated cultures (Figure 4A, 1.5 days). Similarly, HKL from lake sticklebacks displayed the highest respiratory burst activity with *D. pseudospathaceum* antigen stimulation (Figure 4B, 1.5 days). With *D. pseudospathaceum* antigen stimulation, river stickleback, contrary to lake sticklebacks, seemed to reduce their respiratory burst activity after pre-exposures only (5/0), but this was significant only between the pre-exposures only (5/0) groups.

Overall, pre-exposure only to *D. pseudospathaceum* (5/0) increased the capacity for a respiratory burst response in lake sticklebacks, which was abolished by an additional exposure (5/1). River sticklebacks responded rather with a down-regulation of the respiratory burst upon pre-exposure only (5/0) and recent exposure (0/1 and 5/1). However, exposure treatment was not significant in the GzLMs of both, zymosan and *D. pseudospathaceum* antigen stimulation (Table 2) and consequently effects of exposure treatment have to be interpreted with caution. The respiratory burst activity tended to decrease from day 1.5 to day 5, (also in sham treated controls), which might be attributed to the fact that on day 1.5 sticklebacks were sampled straight from the exposure tanks, whereas day 5 fish were returned to their home tanks before sampling, but sampling time point was significant only in the GzLM for stimulation with *D. pseudospathaceum* antigens (Table 2).

### Proliferation of lymphocytes and monocytes

The proliferation of lymphocytes in head kidney leukocyte (HKL) isolates changed significantly with sampling time point and exposure treatment (GzLM, Table 2). As a trend HKL from lake sticklebacks, isolated 1.5 days after the last exposure, higher proportions of proliferating (activated) lymphocytes were observed in sticklebacks with the recent single *D. pseudospathaceum* exposure (0/1 and 5/1), compared to sham exposed controls (0/0) (Figure 5A), but this was not significant with sequential Bonferroni correction. Lymphocytes in HKL isolates from river sticklebacks were even less responsive to the *D. pseudospathaceum* exposures. On day 5 after the last exposure, lymphocyte proliferation was generally lower, which might explain the significant effect of sampling time point in the GzLM (Table 2), but differences between treatments or stickleback origins were not detectable (Figure 5A). In the GzLM of monocyte proliferation, exclusively the interaction of sampling time point, origin and exposure treatment was significant (Table 2). The proliferation of monocytes was elevated in HKL isolates from both stickleback origins in the treatment groups (0/1 and 5/1) with recent single exposure in the sampling after 1.5 days of infection (Figure 5B). Similar to lymphocytes, monocytes did not show responses to *D. pseudospathaceum* exposure after 5 days of infection (Figure 5B). Both, lymphocytes and monocytes showed elevated proliferation (activation) only 1.5 days after the last exposure with *D. pseudospathaceum*, suggesting that activation of these cells in the head kidney is induced only for a relatively short time, upon an acute exposure.

## Discussion

Three-spined sticklebacks originating from a lake with high prevalence of the eye fluke *D. pseudospathaceum* showed higher resistance to the parasite, compared to sticklebacks from a river with low *D. pseudospathaceum* prevalence. In sticklebacks from both origins, a prominent reduction of the infection success of *D. pseudospathaceum* was detected, when the fish had previously been exposed to the parasite. The present data confirm that stickleback ecotypes adapt their immune competence to the parasite infection pressure in their habitat of origin. Furthermore, the present study demonstrates that three-spined sticklebacks acquire immunity to *D. pseudospathaceum*, if they are exposed to the parasite repeatedly.

In a previous study, sticklebacks were pre-exposed only two times with 20 cercariae each and did not acquire substantial immunity against *D. pseudospathaceum*[11]. In the present study, sticklebacks were pre-exposed five times with 20 cercariae, which reduced the infection success of a subsequent exposure by more than 50%. While the total numbers of *D. pseudospathaceum* per eye lens were always higher in river than in lake sticklebacks, the relative reduction of infection success was similar between stickleback ecotypes. This suggests that sticklebacks from the river population, although rarely encountering eye fluke infections in nature, were as capable as lake sticklebacks in developing an acquired immune response against *D. pseudospathaceum*. This was not necessarily expected, as sticklebacks from the two habitat types differ in diversity and allelic composition of their major histocompatibility complex (MHC) class II genes [9,29]. These cellular receptors, which are essential for an acquired immunity against metazoan parasites, have been shown to be responsible for resistance of sticklebacks against the most prevalent species of the respective habitat-specific parasite fauna [30]. Thus, MHC-dependent acquired immunity in the different stickleback populations should be especially adapted to cope with sympatric rather than allopatric parasites. Nonetheless, the relative decrease of susceptibility towards *D. pseudospathaceum* in river sticklebacks after repeated infections in this experiment might be due to MHC-dependent acquired immunity, whereas the different absolute levels of susceptibility in the two ecotypes towards *D. pseudospathaceum* in all treatment groups are more likely due to differences in the innate immune effector mechanisms.

The prominently lower infection success of *D. pseudospathaceum* in lake sticklebacks in this experiment confirms the findings of previous studies [11,13], where lake sticklebacks showed higher activity in innate immune functions. In the present study, lymphocyte proliferation was generally higher upon a recent (1.5 days before) exposure to *D. pseudospathaceum*, but not at day 5 (significant main effect of sampling time point, but not significant with sequential Bonferroni correction) or day 15 thereafter. This is rather in support of a T-helper cell 1 (Th1) induced cytotoxic activity, than a Th2 response, leading to B-cell activation and lymphocyte proliferation (Figure 5A). The observed elevated proliferation of monocytes upon recent exposure is in support of Th1 induced cellular activity (Figure 5B).

In rainbow trout, strong cytotoxic activity against *D. spathaceum* was detected [20]. Rainbow trout immunized with sonicated *D. spathaceum* developed an antibody response, and such immune sera in combination with macrophages enhanced the killing of *D. spathaceum* diplostomules [22]. However, production of antibodies to *D. spathaceum* was only induced upon injection with parasite antigens, but not after exposure to infective cercariae [17]. This suggests, that Diplostomum is able to evade an antibody mediated acquired immune response, when it is using its natural route of infection and is exposed to the immune system only for a short time before it reaches the eye lens.

In the present study, the respiratory burst activity of head kidney leukocytes, a function of innate cellular immunity, which might facilitate killing of the parasite during a cytotoxic response, was recorded. Here the more resistant lake sticklebacks showed generally a higher activity. Furthermore, lake sticklebacks that were only pre-exposed to *D. pseudospathaceum* (Figure 4, day 1.5, 5/0) showed elevated respiratory burst activity. When pre-exposed sticklebacks were additionally exposed (Figure 4, day 1.5, 5/1), respiratory burst activity was reduced. In river sticklebacks, the respiratory burst (Figure 4) tended to decrease after exposure to *D. pseudospathaceum*, and in contrast to lake sticklebacks also in the only pre-exposed (5/0) group. Both, the respiratory burst response to zymosan (r = 0.436) and *D. pseudospathaceum* antigens (r = 0.257) were positively correlated (p < 0.001, n = 446) to the proportion of granulocytes present in head kidney isolates. Taken together, higher respiratory burst responses, presumably mediated by higher granulocyte frequencies, of lake sticklebacks to *D. pseudospathaceum* exposure, might (at least partially) explain, why these fish are more resistant to the parasite. When analysing the transcriptome wide gene expression of lake and river sticklebacks after a first and a second exposure to three parasite species, among those *D. pseudospathaceum*, Lenz *et al.*[12] observed that lake ecotypes showed stronger responses of immune genes, which were mainly representatives of innate immunity. Even after the second exposure, when an acquired immune response would have to be expected, mainly innate immune genes responded and the authors interpreted this as a “reactivation” of innate immunity rather than an activation of acquired immunity [12].

Functional immune parameters of head kidney leukocytes from the present study correspond with the findings on gene expression profiles. Here as well, the (innate) respiratory burst activity of lake sticklebacks was higher compared to river sticklebacks. Repeated exposure to *D. pseudospathaceum* resulted in an up-regulation of respiratory burst in lake sticklebacks but not in an up regulation of lymphocyte proliferation, typical for an acquired immune response.

## Conclusions

The present study confirms that three-spined stickleback ecotypes adapt their immune competence to the habitat specific parasite infection pressure and demonstrates that sticklebacks acquire immunity to *D. pseudospathaceum*, if they are exposed to the parasite repeatedly. Adaptation of immune competence of lake sticklebacks to *D. pseudospathaceum*, as well as higher resistance after repeated exposures to the parasite seems to be mediated by the activity of the innate, but not, or at least to a lesser extend, by the acquired immune system.

## Competing interests

The authors declare that they have no competing interests.

## Authors’ contributions

The project was initiated by MK but both authors (MK and JPS) designed the experiment. MK collected and prepared the infective stages and antigens of *D. pseudospathaceum* and performed the exposures of the sticklebacks. Dissection of fish and parasite screen was done by authors, MK and JPS, and the acknowledged helpers. The immunological assays were mainly performed and analysed by JPS. Both authors have approved the present version of the manuscript.
